# Microplasma Field Effect Transistors

**DOI:** 10.3390/mi8040117

**Published:** 2017-04-05

**Authors:** Massood Tabib-Azar, Pradeep Pai

**Affiliations:** 1 Department of Electrical and Computer Engineering, University of Utah, Salt Lake City, UT 84112, USA; 2 Technology and Manufacturing Group, Intel Corporation, Hillsboro, OR 97124, USA; bpradippai@gmail.com

**Keywords:** plasma devices, atmospheric-pressure plasmas, glow discharge devices, power amplifiers, terahertz switches

## Abstract

Micro plasma devices (MPD) with power gains are of interest in applications involving operations in the presence of ionizing radiations, in propulsion, in control, amplification of high power electromagnetic waves, and in metamaterials for energy management. Here, we review and discuss MPDs with an emphasis on new architectures that have evolved during the past seven years. Devices with programmable impact ionization rates and programmable boundaries are developed to control the plasma ignition voltage and current to achieve power gain. Plasma devices with 1–10 μm gaps are shown to operate in the sub-Paschen regime in atmospheric pressures where ion-assisted field emission results in a breakdown voltage that linearly depends on the gap distance in contrast to the exponential dependence dictated by the Paschen curve. Small gap devices offer higher operation frequencies at low operation voltages with applications in metamaterial skins for energy management and in harsh environment inside nuclear reactors and in space. In addition to analog plasma devices, logic gates, digital circuits, and distributed amplifiers are also discussed.

## 1. Introduction

Plasmas have been extensively studied during the past century [1]. Their applications in large-scale devices for fusion, and small-scale devices in switches are well accomplished and developed. Here, we concentrate on cold plasmas that can be easily generated in a small (<1 mm^3^) volume with moderate electrical powers of less than five Watts and discuss their applications in devices similar to diodes, MOSFETs and digital and analog three-terminal devices with power gains for amplification of signals. Except in distributed plasma devices and in magnetic field sensors, we only consider non-magnetized plasmas. For the most part, we assume that the plasma is quasi-neutral and it is generated at atmospheric pressures that range from 0.6 to 1.1 atmospheres.

Microplasma devices have received a renewed attention in the past 5–7 years owing to their potential applications in harsh environment. Important features that make plasma devices attractive are: (a) their very large off-to-on resistance ratios (10^10^ Ω/0.1 Ω); (b) the ability to conduct very large currents; (c) the ability to operate at very high temperatures (can be as high as 1000 °C); (d) improved operation in the presence of ionizing radiation; (e) the ability to traverse shortest “electrical” distance between their anode and cathode (this property can be used to solve “shortest” path problems) [2]; and (f) the ability to form programmable electrically conducting paths making them suitable for reconfigurable antennas and circuits [3]. MPDs are also being explored in developing chip-scale electron beam accelerators and a 100 GeV electron accelerators has already been demonstrated [4].

Microplasma devices are currently used in displays [5], light sources [6], ionization devices for chemical analysis [7], material processing [8,9,10,11,12], medicine for sterilization without using chemicals [13], and treating skin conditions and wounds to enhance healing [14]. In all these applications, the MPDs are two-terminal devices and behave like switches that are either “on” or “off”.

In addition to the above devices, MPDs with internal controls are reported. Chen and Eden developed a plasma transistor very similar in operation to a bipolar junction transistor (BJT), consisting of an electron emitter [15]. They were able to increase the conduction current by four fold by biasing the electron emitter by −20 V. Wagner et al. from the same group, developed a plasma BJT that consisted of a hybrid plasma-semiconductor interacting device [16]. The plasma BJT exhibited a voltage gain of 27 and was able to turn off the device (plasma) with a mere 1 V of emitter-base voltage. The active gain in these devices was produced by the semiconductor part of their structure that acted as charge injector upon optical illumination or when their surface P–N junction was forward biased.

**Figure 1:**
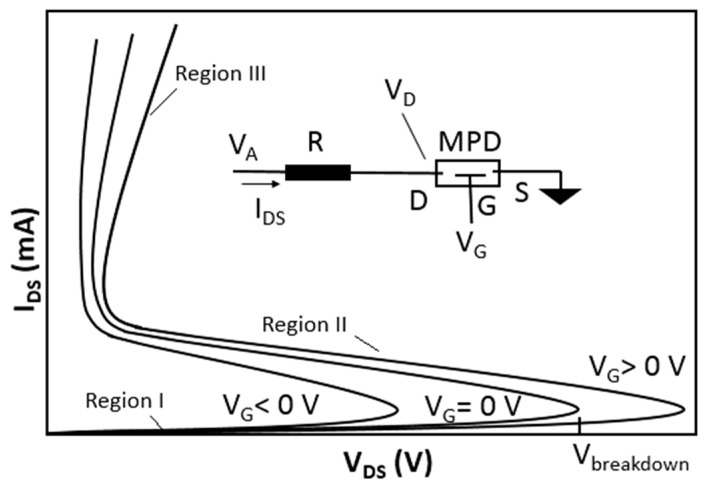
Schematic of current versus voltage I–V characteristics of a three-terminal microplasma device.

Devices that rely on the modulation of carriers in the plasma without using any semiconductor structure are also reported. Yuan et al. developed a microplasma transistor that relied on a gate electrode field effect to modulate the ionic concentration in a radio frequency (rf) plasma, which in turn modulated the plasma current [17]. They also demonstrated the feasibility of operating a plasma device under harsh environment involving high temperature and ionizing radiation inside a nuclear reactor. In these MPDs the operation voltages were above a few hundred volts because of their ionization gaps of larger than 10 μm. Here we discuss a three-terminal MPD where similar to [17], a third terminal (i.e., gate) is used to “modify” the plasma generation voltage and current through the other two terminals (i.e., drain and source) as schematically shown in Figure 1. These current versus voltage (I–V) curves are composed of three regions: in region I the current is very small and is mainly due to very small conduction due to random gas ionization; in region II the current increases without bound due to the gas breakdown and impact ionization (at *V_DS_* = *V_breakdown_*); and in region III the ionized gas behaves like a resistor and the current increases nearly linearly as a function of the voltage. The role of the gate voltage is to modify the breakdown voltage and the plasma current. The gate voltage can affect the ionization process by changing the concentration of the secondary electrons in the channel. The gate voltage can also change the boundary space-charge regions and the effective ionization path or active channel’s effective cross section.

**Figure 2:**
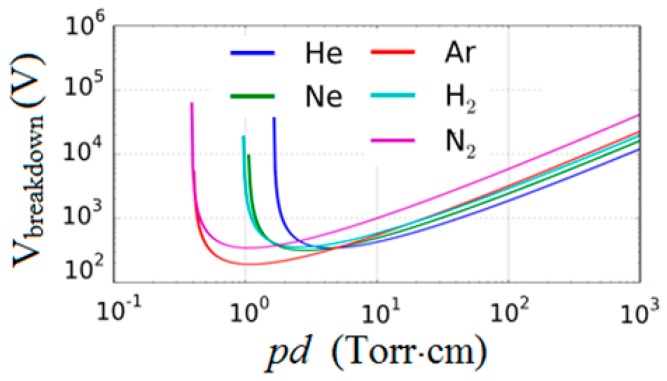
Paschen curves for five different gases of importance in MPDs as a function of gap distance at 760 Torr.

Classically, the gas breakdown voltage is determined by the Paschen curve shown in Figure 2 [18]. As the gap distance is reduced below 10 μm at 1 atmosphere in nitrogen, the Paschen curve predicts very large breakdown voltages, which is experimentally shown not to be accurate. The ion-assisted field emission takes over in this regime and lowers the breakdown voltage considerably [19,20,21,22,23,24]. If it were not for the ion-assisted emission process, it would have been impossible to realize MPDs with single digit breakdown voltages.

The condition for generation of self-sustained plasma is governed by the Townsend’s breakdown criterion that defines the condition for the electrical breakdown of a gas. Extrapolation of the breakdown criterion leads to the Paschen’s law (Equation (1)) that relates the breakdown voltage of a gas to its pressure and electrode separation. Paschen law is expressed as [14]:

(1) $$V_{BD}=\frac{Bpd}{\ln\left(Apd\right)-\ln\left(\ln\left(1+\frac{1}{\gamma_{i}}\right)\right)}$$

(2) γi[eApd {exp(−BpdVBD)}−1]=1

where $\gamma_{i}$ is the secondary emission coefficient for ions, *A* and *B* are two empirical coefficients that are found to be nearly constant over a range of voltages and pressures for any given gas, *p* is the pressure, *d* is the gap size, and *V_BD_* is the breakdown voltage. For argon and tungsten electrodes, *A* is 10.20 Pa^−1^·m^−1^, *B* is 176.27 Pa^−1^·m^−1^ and $\gamma_{i}$ is 0.095.

From Equation (1) it can be seen that the breakdown voltage can be modified by changing $\gamma_{i}$: $\frac{\partial V_{BD}}{\partial \gamma_{i}}=\frac{Bpd\gamma_{i}/\ln\left(\frac{1}{\gamma_{i}}\right)}{{\left[\ln\left(Apd\right)-\ln\left(\ln\left(\frac{1}{\gamma_{i}}\right)\right)\right]}^{2}}$, with 1/$\gamma_{i}$ > 1. A convenient method to modify $\gamma_{i}$ is by using electric field effect to modify secondary electron concentration by electrostatically attracting/repelling them by a gate electrode as exploited in our work reported here.

**Figure 3:**
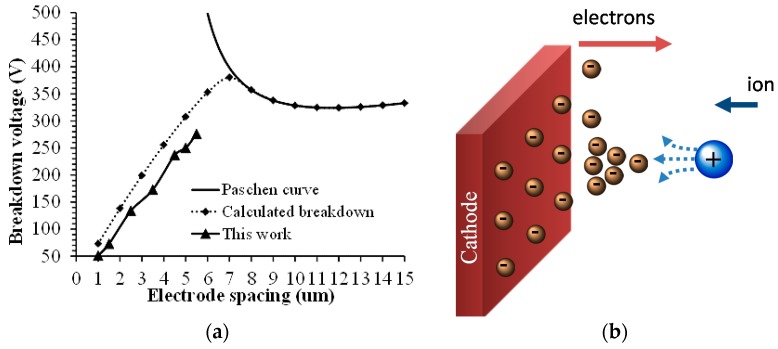
Modified Paschen curve for air. (**a**) The ion-assisted field ionization results in complete departure from the classical Paschen curve for small gap distances of less than 10 μm in one atmosphere in most gases [24]; (**b**) Schematic representation of electron emission at cathode due to electric potential of an approaching ion [25].

When we decrease the gap distance below around ten times the electron mean free path, the electrons generated by the ionization process do not gain enough energy to initiate the avalanche breakdown process. Thus the breakdown voltage increases as indicated by the above equations and shown in Figure 2 below *pd* ≈ 2 Torr·cm. Recent experiments, however, have shown that the breakdown voltage decreases monotonously for small gaps [19,20,21,22,23,24] that are less than 10 μm in 1 Atmosphere in air. The cause of this behavior is attributed to ion-enhanced field emission that occurs as the positive ions approach the negative electrode (cathode). In gas breakdown, the electron emission from the cathode occurs as a result of the energetic ions bombarding the cathode surface and knocking out the electrons. At small inter-electrode gaps (<10 μm), the electron emission is also affected by the cathode field emission, which is enhanced by the electric potential of approaching ions as shown in Figure 3b. At small gaps, the yield of the ion field-assisted electron emission far exceeds the electron emission from collisions with neutrals. Thus, the field-assisted emission becomes the primary ionization mechanism at small gap [23,24]. Tirumala and Go [22] used an approach that modified the existing Paschen’s law to accommodate the field-emission. They added an electron emission coefficient ($\gamma^{\prime }$) to the existing equation to obtain a modified Paschen’s law (Equation (2)) that applied to any given inter-electrode gap and gas pressure. Our work extends the modified Paschen’s law to accommodate the effect of gate field-effect. The model was first curve-fitted to match the experimental breakdown voltages [22,24].

(3) (γi+γ′)[eApd exp(−BpdVBD)−1]=1

where the ion enhanced field-emission coefficient $\gamma^{\prime }$, is given by

(4) γ′=∫0R(2πrdrq)∫0Tdt[AFNE(r,t)2φt2(y) ]exp(−BFNφ32v(y)E(r,t))

and the net electric field at the cathode E(r,t) is given by

(5) E(r,t)=(βEA)+q2πε0L0−bEAt[(L0−bEAt)2+r2]32

where r is the linear dimension of the cathode, $A_{FN}$ and $B_{FN}$ are Fowler–Nordheim field-emission constants, E(r,t) is the electric field at the cathode due to the approaching positive ion, φ is the work-function of the cathode metal, t(y) and v(y) are functions discussed in [23], β is the field-enhancement factor due to asperities on the cathode, $E_{A}$ is the electric field between the cathode and anode due to applied voltage and is equal to $V_{b}/d$, $L_{0}$ is the distance from the cathode at which the ion is created, b is the ion mobility and *t* is the time. The value for $\gamma_{i}$, R and $L_{0}$ are 0.0075, 125 nm and 25 nm respectively as given in [18]. The value of β depends on the surface condition of the cathode. For a perfectly smooth surface, β = 0. In practice β is around 100 due to unavoidable surface asperities. In our work, we used β as the curve-fitting parameter to match the model closely with the experimental results.

According to the modified Paschen curve model, the breakdown voltage in the small gap regime (gap < 10 μm at 1 Atmosphere) is linearly proportional to the gap size as shown in Figure 3 and, as expected, it becomes zero for zero gap distance.

## 2. Plasma Carrier Dynamics and Concentrations

In addition to the gate-control mechanism discussed above, carrier densities and mobilities are also required to design microplasma switches. The density of gas molecules in 1 atmosphere is around 10^19^ cm^−3^. In most small scale plasmas, the density of electrons and their corresponding positive ions range between 10^13^ and 10^16^ cm^−3^. Mean free path of gas molecules in one atmosphere at room temperature is around 70–90 nm. The mean free path of electrons in atmospheric plasma is around 0.5 μm.

**Figure 4:**
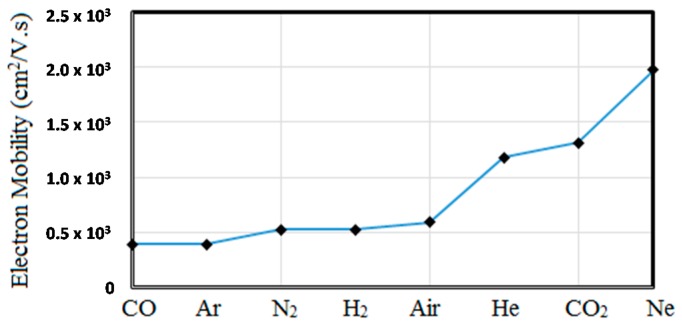
Electron mobility in plasma of different gases used in MPDs.

The electron mobility (*μ_e_*) relates electron’s drift velocity (*v_d_*) to the applied electric field (*E_a_*): *v_d_* = *μ_e_E_a_*. It can also be shown that *μ_e_* = *e/mυ_en_* where *e* is the electronic charge (1.6 × 10^−19^ Coulomb), m is its mass and *v*_en_ is the frequency of electron-neutral collisions. *v_en_*/pressure is around 5 × 10^9^ s^−1^·Torr^−1^ in most gases and in 1 atmosphere it becomes *v_en_* ≈ 3 × 10^12^ Hz. Figure 4 shows electron mobility in atmospheric plasmas in some gases of interest for MPDs. It can be shown that *μ_e_*/pressure is also nearly constant and ranges from 0.4 to 2 in most gases [26].

Carrier mobility is inversely proportional to the carrier mass. Ions being much heavier than electrons, have much smaller mobility and for all practical purposes can be ignored in MPDs. It is interesting to note that the electron mobility in atmospheric plasma is comparable to the electron mobility in silicon in Figure 4. By reducing the plasma pressure to 1 Torr, the electron mobility is enhanced by ×760 making it higher than electron mobility in graphene and other high performance 2D and bulk semiconductors at room temperature.

**Figure 5:**
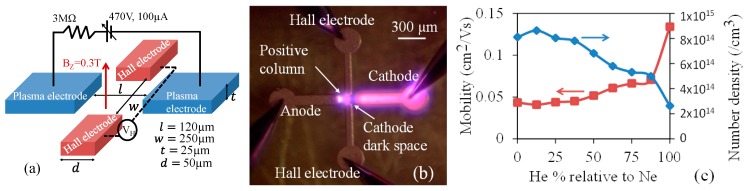
(**a**) Experimental setup for the Hall measurement. (**b**) Optical image of the Hall measurement electrodes during operation. The Hall electrodes are in contact with the positive column and the cathode dark space. (**c**) Estimates of ion mobility and density obtained from the measured Hall voltage for a mixture of He and Ne gas in different proportions. All figures are reproduced from [25].

Plasma carrier mobility in micro-devices can be measured using the time of flight, conductivity, and Hall measurements similar to semiconductors. Figure 5a shows schematic of a plasma Hall device where charge carriers of the plasma, generated between the left and right electrodes, are diverted by an applied magnetic field through the Lorentz force (*q**vxB***) and sensed by the top and bottom electrodes. The signal strength depends on carrier concentration if measured in the open circuit voltage mode. Figure 5b shows the image of the Hall device with plasma. In this case, the cathode dark region [27] is near the Hall electrodes.

It should be noted that the Hall electrodes shown in Figure 5a should be covered with a dielectric layer with large breakdown voltage to prevent arching or plasma generation between these electrodes and the plasma electrodes. In this case, the Hall electrodes capacitively sense the charges that are diverted by the Lorentz force. Alternatively, the Hall electrodes can also be “balanced” to reside at a virtual ground node between the two plasma electrodes. In this case, they can be used to draw small current for dc measurements.

A plasma contains moving electrons and ions that in the presence of the external magnetic field, experience the Lorentz force. The plasma has different regions that can be seen as striations of glowing and dark regions with different ionic and electronic charge densities [27]. The glowing region close to the anode is the positive column which is quasi-neutral. The dark space near the cathode is the cathode dark space, where most of the ionization occurs and it is positively charged. The response of electrons or ions to the magnetic field can be separated from each other by placing the Hall electrodes close to the anode or cathode respectively. At small electrode gaps and higher pressures, the gap is mostly filled with the positive column that transforms to the cathode dark space as it approaches the cathode, as evident in Figure 5a. Due to the positive space charge in the cathode dark space, our Hall voltage measurements will reflect ion mobility and density. The measurements are made with a mixture of He and Ne at different ratios to observe the effect of different gases on carrier mobility and charge density.

The Hall electric field obtained for different gas compositions are used to calculate the ion mobility and its density using the Lorentz force [25,28,29]:

(6) $$E_{H}≅-v\times B_{Z}=\frac{B_{Z}\times J_{X}}{q\times n}$$

(7) $$\mu =\frac{v}{E_{X}}\;\mathrm{and}\;E_{X}=V_{X}/l$$

where $E_{H}$, v, $B_{Z}$, $J_{X}$, *V_X_*, *E_X_*, q, n, μ and l are the Hall electric field, ion velocity, traverse magnetic field, longitudinal current density, longitudinal voltage, longitudinal electric field, electronic charge, ion density, ion mobility and plasma electrode separation, respectively.

Electron lifetime in the plasma is very short and is approximately 1*/v_en_ ≈* 0.3 ps. Another important plasma parameter is the Debye length that signifies the electrostatic screening length and it is given by: $\lambda_{D}=\sqrt{\frac{\epsilon_{p}kT_{e}}{e^{2}N_{e}}}$, where *ε_p_* is the plasma permittivity, *N_e_* is the electron concentration, *e* is the electron change, *k* is the Boltzmann constant, and *T_e_* is the electron temperature. For electron temperature of 10,000 K, density of 10^14^ cm^−3^ the Debye length is *λ_D_ ≈* 1 μm. A grounded electrode immersed in the plasma will be surrounded by positively charged space charge region that is roughly one Debye length thick.

## 3. Three-Terminal MPDs

Here, we discuss MPDs with internal current/voltage control [25,29,30,31,32,33,34,35,36]. We note that surface effects are dominant in scaled MPDs [37]. Electrons with kinetic energies well above a few eV pass through most dielectric and semiconducting materials and leak to the electrical ground. Ions, on the other hand, are contained and do not leak to ground. This situation creates a space charge region near any boundary whether it is conducting or insulating. The bulk of the plasma remains quasi-neutral and only near boundaries there are space charge regions about a Debye-length thick. The voltage drop across the quasi-neutral bulk plasma is very small. The voltage drop across the plasma is mainly concentrated across space charge regions connecting the plasma to electrodes and boundaries. This is also the situation in semiconductors. In semiconductors the equilibrium space charge widths are fixed depending on doping concentrations and contacting metal workfunctions. In plasmas, one can change the Debye length by changing the ionization of the plasma and gas pressure that change the electron concentration in the plasma (*N_e_* in the equation for *λ_D_*).

**Figure 6:**
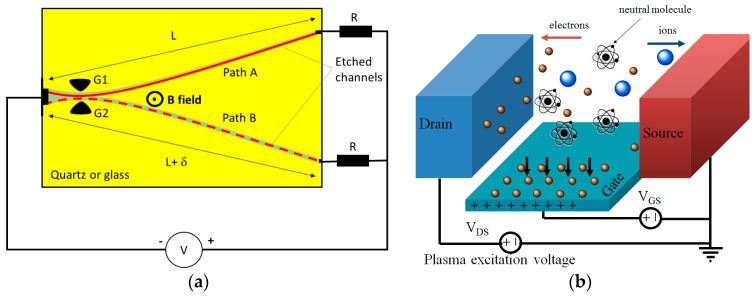
Current control mechanisms used in MPDs. (**a**) A perpendicular magnetic field B is used to switch the plasma between two equivalent paths A and B. (**b**) A positive gate bias sinks the electrons away from the channel region increasing the channel breakdown voltage and a negative gate bias would push electrons into the channel region reducing the channel breakdown voltage [25].

As in semiconductor devices, it is possible to control the flow of electrons in MPDs using electric field effect as well as charge injection as schematically shown in Figure 6 MPD current can also be controlled by changing the plasma path (Figure 6a). In this mode of operation, the plasma path can be changed using a gate field effect or by using an external magnetic field.

## 4. Metal Oxide Plasma Field Effect Transistors (MOPFETs)

**Figure 7:**
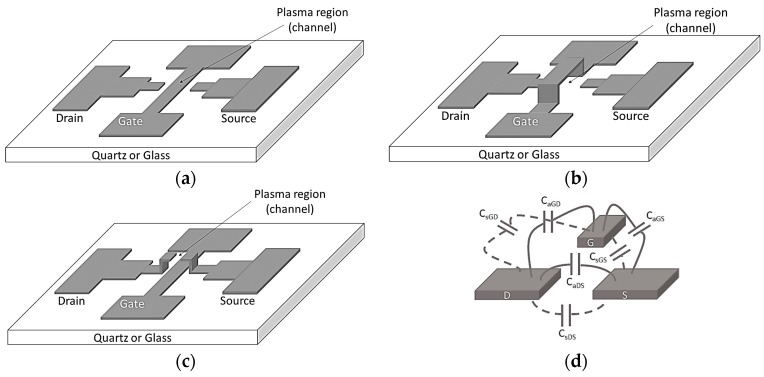
Schematics of different MOPFET geometries reported in the literature: (**a**) Planar MOPFET; (**b**) gate over the drain-source region; (**c**) drain and source electrodes over the gate region; and (**d**) different capacitances between the drain, source and gate electrodes.

In this work, we focus on devices that use electric field effect (gate voltage) to modulate plasma current. Microfabricated plasma devices can be divided into lumped and distributed architectures with planar and 3D geometries. Distributed devices have extended geometries where the plasma interacts with the device in multiple points such as in traveling wave tubes. Lumped devices are “point” devices like MOSFETS. 3D and 2D lumped MPDs are schematically shown in Figure 7 without a possible gate dielectric. The planar geometry (Figure 7a) was tried first and surprisingly worked well but the raised drain-source geometry (Figure 7c) showed the best performance. In all cases, the drain, gate, and source electrodes are coupled to each other capacitively through the substrate as well as through the air as shown in Figure 7d. In the raised electrode geometries (Figure 7b,c) the parasitic substrate capacitance is smaller than in the planar geometry (Figure 7a). The substrate parasitic capacitance and the capacitance through the air are connected in parallel and share the same voltage. Smaller substrate capacitance results in smaller substrate leakage current and results in less damage due to charge injection. Raised electrodes are more efficient in gas ionization due to their larger effective surface area.

**Figure 8:**
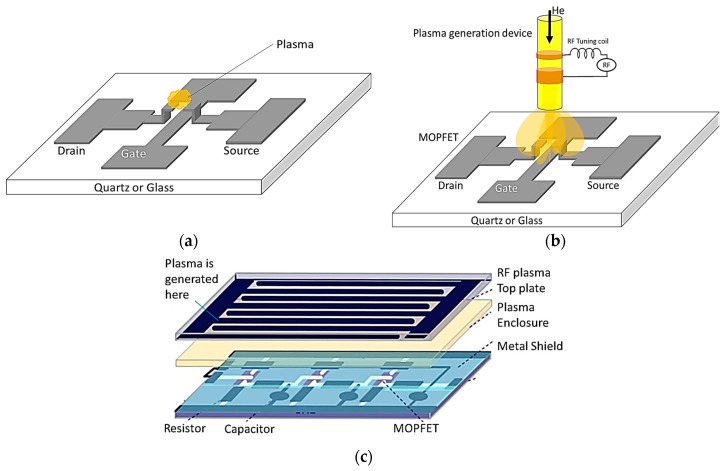
Schematics of: (**a**) self-generating plasma MOPFET; and (**b**) separate medium device; (**c**) Schematic of a separate-medium integrated circuit with MPDs [32].

Two different methods can be used in active plasma devices. In self-generating devices, the electrodes that constitute the active part of the device also generate the plasma (Figure 8a). In separate medium devices, the plasma is generated using a separate set of electrodes and diffuse to the device region (Figure 8b). In both cases, it is possible to envision integrated plasma circuits as schematically shown in Figure 8c.

We have developed many different MPDs during the past four years [17,25,28,29,30,31,32,33,34,35,36] and we are currently developing distributed MPDs for amplification of terahertz signals using plasma interaction with periodic structures [29,38,39]. Here we demonstrate the operation of relatively low voltage sub-5 µm gap microplasma transistors that operate at microwave frequencies based on the device geometry schematically shown in Figure 8c. The small gap lowers the device turn-on voltage (gas breakdown voltage) by operating it in the sub-Paschen regime. Moreover, the gate capacitance is minimized by optimizing the device geometry to achieve drain current modulation speeds of 7 GHz through gate field-effect, which is comparable to MOSFET speeds.

The MOPFET device structure is schematically shown in Figure 8 and consists of three regions of source, drain and gate similar to MOSFETs. The drain and source regions are separated by a gap where plasma gases reside. The gate is placed between the drain and source and is situated out of the source-drain plane by a stand-off distance. The conducting channel for drain-source current is provided by the breakdown of gases and generation of conducting plasmas in gap. Application of a voltage on the gate modifies the charge density in the plasma through the gate field effect that modulates the drain-source current. Plasma can be generated by dc or microwave voltages. DC excitation has inherent ion-sputtering problem that damages the electrodes and cannot support large currents as seen in our earlier work [17,33]. Using microwave voltages for plasma generation improved the device life significantly. The dynamic response of the device is determined by the gate-source voltage. The drain-source current can be modulated by the gate voltage over a wide range of frequencies from dc to a few GHz. However, since the ions respond to low frequency signals, it is desirable to operate the device at gate excitation faster than few tens of kilohertz to prevent sputtering damage to the gate electrode.

In MPDs, the plasma is in a confined space with dimensions comparable to the mean-free path of its electrons (~0.5 μm) and 5–10 times larger than the mean free path of gas molecules (70–100 nm). Thus, the boundary effects can dominate [37]. Boundaries sink the electrons and produce surface space charge regions that can be a large fraction of the plasma volume in these devices. In RF microplasmas, the electrons are accelerated in opposite directions when the electric field switches sign. Thus, the mean distance electrons travel in the positive or negative part of the cycle is another length scale of importance.

Due to the heavier mass of ions, they fail to follow the oscillations and remain practically stationary. At the onset of plasma, the electrical resistance of the gas significantly reduces causing a large current to flow through the ionized gas. The current is limited only by an external resistor or built-in limit of the microwave source. The onset of plasma is accompanied by a visible glow, indicating a glow discharge. Although arc discharge is also accompanied by a visible glow, the current required to produce an arc discharge is very high and beyond the capacity of the voltage sources used in this work. The operating currents in the devices tested here are less than 500 μA.

The electric field necessary to cause ionization of a gas depends on the gas species, pressure, the distance between the electrodes and the nature of the nearby boundaries [25,37]. In our devices, the presence of the gate electrode inside the plasma (Figure 8b) enabled us to actively sink the nearby secondary electrons by applying positive gate voltages or injecting electrons into the plasma by applying negative gate voltages. Modifying the electron concentration in the plasma leads to modification of its ionization rate: positive gate voltage increases the breakdown voltage while negative gate voltage reduces the breakdown field. Helium gas (99.5%) is used in this work due to its relatively low breakdown voltage at atmospheric pressure.

**Figure 9:**
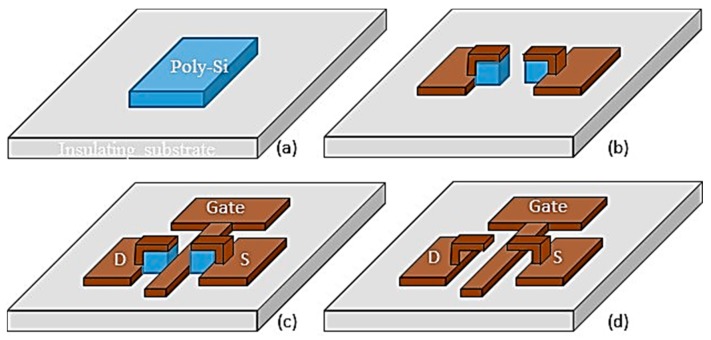
Schematic of the device fabrication. Polysilicon was used as sacrificial material and was preferentially etched away using XeF_2_. (**a**) The first step is to deposit and pattern the sacrificial polysilicon; (**b**) The second step is to deposit and pattern the source-drain metal (W or Cu); (**c**) The third step is to deposit and pattern the self-aligned gate metal; (**d**) The last step is to remove the polysilicon to free channel regions of the drain-source electrodes.

The fabrication process was designed to produce a self-aligned gate electrode as schematically shown in Figure 9. A good gate alignment is necessary to reduce parasitic capacitances between gate-drain and gate source regions. The process starts with the deposition and patterning of a 0.5 µm thick layer of poly-Si that defines the stand-off distance between the drain-source and gate electrodes. A 0.5 µm thick layer of TiW is then sputtered and pattern to define the drain-source electrodes. The underlying poly-Si is patterned along with the drain-source to define the gate area. A 0.2 µm thick layer of TiW is then sputtered and patterned to form the gate. The sacrificial poly-Si is then etched away using XeF_2_.

The fabrication process involved two critical steps. The first one was to ensure a low stress deposition of the 500 nm thick TiW. The stress developed in the sputtered film depends on the chamber pressure during deposition and the dissipation of heat generated during sputtering. In this work, we customized the deposition power and time. Interval deposition was found to develop significantly less stress compared to continuous deposition. Sputtering at 200 W power with deposition and rest periods of 5 min each produced low stress films for thickness up to 2 µm.

**Figure 10:**
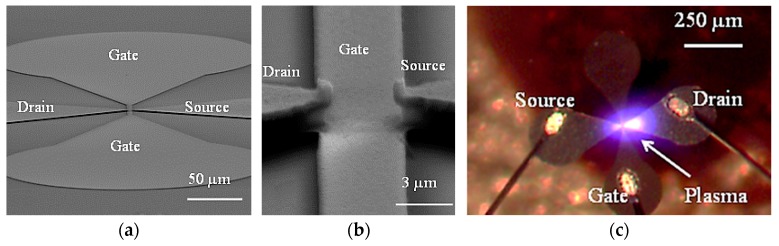
(**a**,**b**) SEM micrographs of the device. The small overlap are between gate and source/drain electrodes reduce the gate capacitance and help achieve higher switching speeds; (**c**) Optical image of the packaged device during operation with rf excitation. The devices were wire-bonded, packaged and sealed with a Plexiglas plate. Helium was continuously supplied to the package through a tubing [25].

Figure 10 shows the scanning electron microscope (SEM) and optical images of the MOPFET. The released devices are wire-bonded to a hybrid package and sealed using a Plexiglas plate. Helium is continuously flown into the package through tubing. Since the package is not hermetically sealed, the pressure inside the package remains at atmospheric pressure.

### MOPFET Characteristics

**Figure 11:**
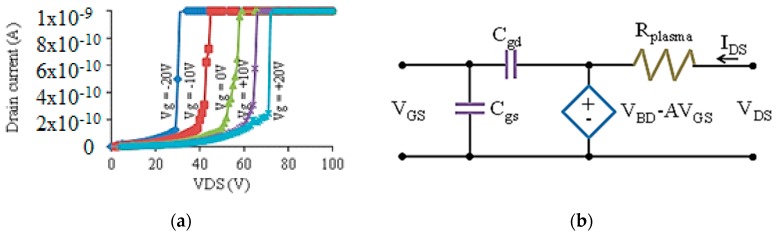

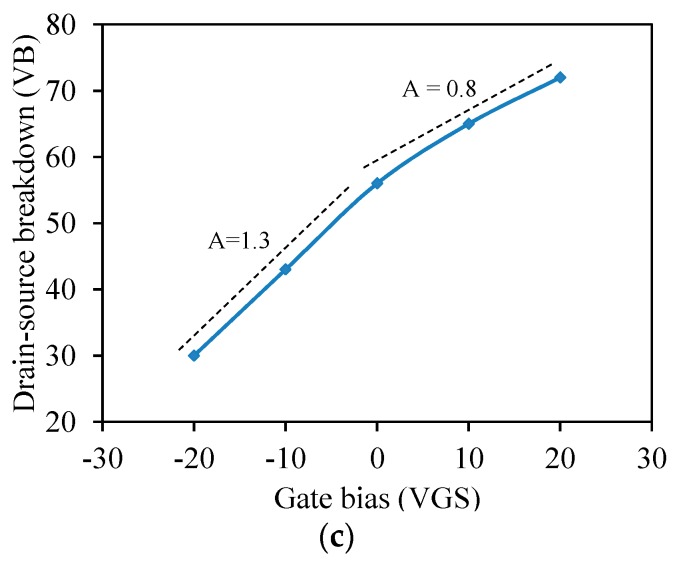
(**a**) I-V characteristics of a 1 μm gap MOPFET. (**b**) A simple two port hybrid-pi model of the MOPFET. The constant voltage source plays a central role in the model and characterizes the accuracy of gas breakdown voltage. (**c**) The voltage gain as a function of gate bias clearly showing the gate field effect [25].

*MOPFET DC Characteristics:* The dc switching characteristics of the MOPFET shows detailed information of the breakdown mechanism and the gate control as reported in our earlier work [17,24]. The breakdown voltages were in the range 30–70 V and were smaller by a factor of 5 compared to other work [17,32]. The low breakdown voltage was achieved by ion-enhanced field emission which takes effect for inter-electrode spacing less than 10 μm. Although the physics of the breakdown mechanism is different for dc and rf excitations, the electrical characteristics of the device remained similar. The main difference between dc and rf operations was in the conduction mechanism. The current conduction in rf excitation is almost entirely due to electrons, whereas dc excitation generates electronic and ionic currents. The ionic current causes sputtering of the cathode material and causes severe irreversible damage in these MOPFETs due to the small cross-sectional area. To prevent electrode erosion, the dc characterization was performed with currents less than 5 nA. Figure 11a shows the dc switching data for a 1 μm gap MOPFET.

The gate bias increases (decreases) the drain-source breakdown voltage for positive (negative) voltages. The output behavior of the MOPFET is different from a MOSFET. A MOSFET acts as a constant current source for a given gate bias in the saturation mode, but the MOPFET is a constant voltage source for a given gate bias. This difference is due to the nature of the gas breakdown. The MOPFET operates in the normal glow discharge mode. In this mode, the voltage across the drain-source remains constant over a wide range of currents and is roughly equal to the breakdown voltage. The resistance is mostly due to the collisions of electrons with ions and neutral gas molecules. The switching behavior can be represented by a simple model as shown in Figure 11b. The dependence of breakdown voltage on the gate bias is represented by the expression for the controlled voltage source where *V_BD_* is the breakdown voltage. The amplification factor “A” depends on the stand-off distance between the gate and source/drain and the carrier mobility.

*MOPFET Gain Mechanisms*: In MOPFETs, the primary mechanism that enables the gate voltage to control the drain source breakdown voltage is the effect of gate electric field on the concentration of the secondary electron density. Secondary electrons are primarily responsible for the avalanche breakdown and by reducing (or increasing) their concentration, the breakdown voltage can be increased (or reduced). In our microplasma devices, the device boundaries are very close to the active region of the device and small changes in the gate voltage can cause a large change in the electrostatic potential seen by the quasi-neutral plasma. Moreover, the electrons are quite energetic (>4 eV) and can be transmitted through most dielectric boundaries. When the gate is positively biased, it readily absorbs the nearby electrons and reduces the concentration of the secondary electrons in the channel. The result is that the drain source voltage has to be increased to maintain the channel plasma. Conversely, when the gate is negatively charged, it repels the electrons that otherwise would be absorbed by the gate electrode and consequently it increases the concentration of the secondary electrons in the channel reducing the plasma breakdown voltage. Figure 11c clearly shows the effect of positive and gate voltage on the breakdown voltage of the MOPFET. For negative *V_g_*, we consistently observe lower *V_B_* while positive gate voltages consistently result in larger *V_B_*s.

Other gain mechanisms are also possible. In addition to modifying the secondary electron concentration, the gate voltage can also change the boundary space-charge regions and the effective ionization path or active channel’s effective cross section.

**Figure 12:**
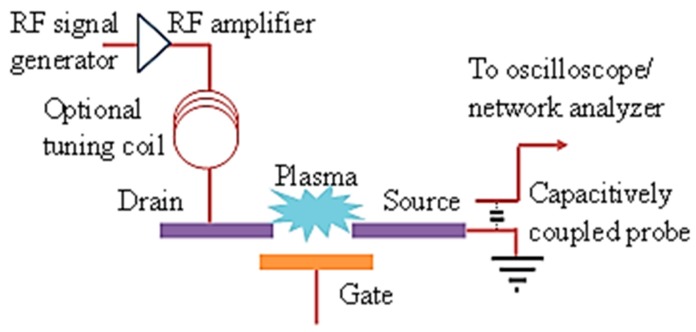
Schematic of the RF measurement experimental setup. The MOPFET device is shown in Figure 10 [25].

*MOPFET RF Characteristics*: RF characterization of the device was done in two stages, each showing the generation of plasma current and the effect of gate modulation at microwave frequencies respectively. The device switching is detected by the drain-source current as shown in the experimental setup in Figure 12. Plasma is first generated by applying voltage between drain and source. To impedance match the rf source and the MPD, a tuning coil was used in series with the drain-source electrodes that constitute a capacitor. At the onset of ionization, large current flows through the drain-source electrodes. The current can be detected by the voltage drop across a small load resistor connected in series with the drain-source, either with an oscilloscope or a network analyzer. In the event of the drain-source electrodes shorting through the gate, the large voltage from the rf amplifier will completely appear across the network analyzer/oscilloscope and damage it. The problem is mitigated by capacitively coupling to the drain-source current using an auxiliary probe. The plasma shown in Figure 10c was sustained by the smallest rf voltage required to generate plasma and, hence, it covers a very small area. For larger rf voltages, the plasma spread out to a wider area covering the source-drain electrodes. The device was tested for up to 20 h of continuous operation with no significant damage to the electrodes.

**Figure 13:**
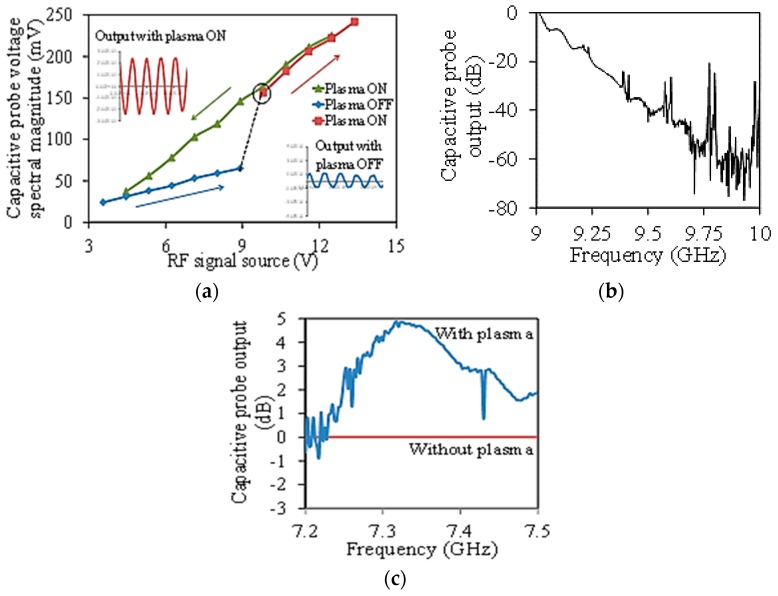
(**a**,**b**) The switching response of the drain-source current for 937 MHz and 9–10 GHz excitation, respectively. The peaks in (b) indicate plasma generation. This experiment was conducted to test the response of plasma to higher frequencies. The gate electrode was not used in both cases [24]; (**c**) The drain current modulation by gate. The effect is greater around 7.3 GHz due to the lower impedance of the lumped inductor-capacitor (LC) components [25].

Plasma current was measured at frequencies in the range of 100 MHz–10 GHz. Figure 13a,b shows the plasma current at 937 MHz and 10 GHz, respectively. The response at 937 MHz was captured in an oscilloscope and the 10 GHz response was measured using a network analyzer since it was above the frequency response of the oscilloscope. The response in Figure 13a showed complete details of the plasma switching. At smaller excitation voltages, the output signal was small with a gradually increasing slope up to 8.9 V (blue line). The small signal detected below plasma turn-on was due to the capacitive coupling. Plasma was generated at 9.8 V indicated by a sudden three-fold increase in the detected voltage. Increasing the excitation voltage increased the output current further that is shown by a steeper slope (red line). The slope was steeper in the plasma ON region due to the smaller resistance of the plasma. There was also a hysteresis observed in the plasma switching while sweeping the voltage in the reverse direction indicated by the green line. This behavior was expected and is similar to that observed with dc excitation [17].

Figure 13b shows plasma current switching near 10 GHz. The plasma power reached a maximum around 10 GHz showing a roughly 30 dB difference in power between ON and OFF states (Figure 13c). The frequency selective behavior is due to the frequency response of the total impedance of the device along with the cables. Practically it is possible to switch plasma at any frequency by varying the impedance or by applying higher excitation power.

For transistor characterization, the drain-source was excited by a slightly lower frequency (600 MHz) rf signal compared to the higher frequency (7–10 GHz) gate excitation signal to avoid unnecessary interference in the detected signal. The drain-source power needed to establish the plasma was fixed at 2 W. The gate signal was limited to 0.1 W. Figure 13c shows the data for transistor operation. The gate modulated the drain-source current by varying the electron density in the plasma. As mentioned earlier, the drain-source plasma was established by a 600 MHz current. Application of the gate bias was expected to modulate this current by the gate signal. This is clearly shown in Figure 13c by the 5 dB offset in the detected current with and without plasma at 7.3 GHz. The gate modulation also shows a frequency selective behavior just like the response in Figure 13b due to the frequency response of the gate-source impedance.

The modulation speed obtained here is 6 orders of magnitude greater than reported by similar devices in the past [17]. The higher speed was due to low gate-source/drain capacitance achieved through smaller overlap region between the self-aligned electrodes and lower parasitic capacitance obtained from the use of glass substrate. There are two important parameters that need to be improved to make this device suitable for practical application. First, the bandwidth of the device is too small for many applications. Second, the effect of gate modulation should be increased for switching applications. It is to be noted that the devices tested in this work were not optimized. The bandwidth and gate modulation can both be improved by optimizing the geometry and impedance matching the device.

## 5. Digital Plasma Devices and Logic Gates

Logic gates using plasma-linked devices (μPD) were demonstrated in the past [34]. The space charge around a microplasma was used to lower the breakdown voltage of a nearby device by 20–40 V. This mechanism was used to establish electrical connection between neighboring microplasma devices without the use of metallization traces. The decay lengths of the space charge were in the range of 178–400 μm depending on the type of gas used. Plasmas can be used to connect devices in three dimensions and their decay constant can be adjusted using pressure, boundary conditions, and gaseous species. Universal gates including OR, AND, NOT and XOR and computer sub-circuits such as 1 bit adders were designed and characterized using plasma-linked devices. In our work presented here, the switching was completely based on the effect of space charge that electrically connects μPDs to form logic gates *without* any metal interconnects. Once the plasma is turned off, the functional electrical link between the input and the output is completely lost. Moreover, different logical operations can be performed using identical devices and inter-device distances by simply changing the plasma characteristics (density, temperature, and spatial decay constant) electronically. This would allow a user to dynamically program the functionality of μPD circuits in real-time.

**Figure 14:**
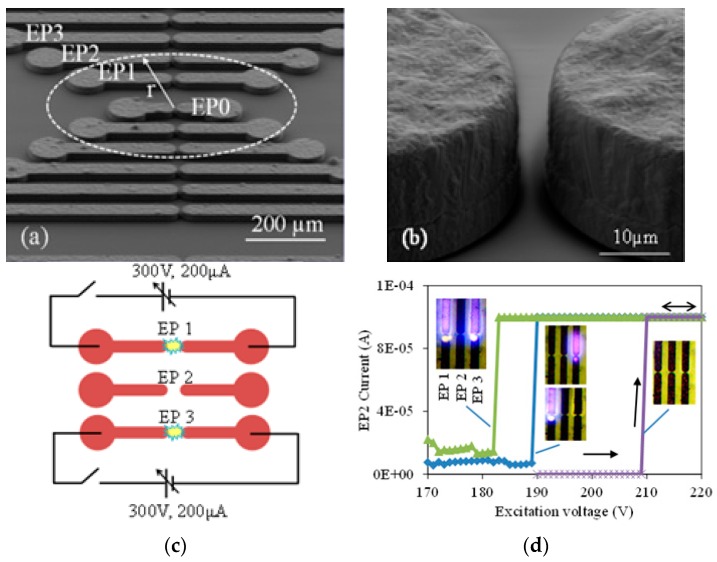
SEM micrograph of devices used for logic gates: (**a**) the adjacent devices are separated by 100 µm; and (**b**) a close-up of one of the electrode pairs. The electrode configuration used to determine the effect of space charge on breakdown voltage of nearby device (**c**); and the experimental results (**d**). Inset shows optical images of the device during operation. All figures are reproduced from [35].

To realize logical operations, μPDs were placed in close proximity to each other so that when a device was turned on, the nearby devices’ breakdown voltage was lowered by the presence of the space-charge produced by the adjacent device. The logic states of the input and output depended on the plasma being ON or OFF. When the plasma is OFF (ON), we obtained the logic “0” (“1”) state. The devices were fabricated using electroplated Cu electrodes with a separation gap of ~5 µm (Figure 14a,b).

The space-charge region at the boundary of the plasma is similar to the Debye sheath that separates the charge-neutral inner plasma region from the outside. Under dc excitation, plasma develops a positive space charge boundary due to accumulation of heavier ions. This positive space charge induces an opposing negative charge through electrons in the surrounding neutral gas region. The effect of these seed electrons on the breakdown voltage of a central device EP0 in Figure 14a was determined while generating plasma in nearby devices EP1–EP3. All measurements in this work were carried out at atmospheric pressure in He. The current was limited to 100 µA in most of our measurements to minimize damage to the cathode from ion sputtering. The breakdown voltage for EP2 was 210 V when both EP1 and EP3 were OFF (Figure 14d). Turning either EP1 or EP3 ON lowered the EP2 breakdown voltage to 190 V. The breakdown voltage was lowered further to 182 V when both EP1 and EP3 were ON.

The space charge surrounding plasma was characterized by measuring the conductance of the gas surrounding the plasma electrodes at distances varying from 100 to 500 µm. The charge density (*C_p_*) as a function of distance can be calculated using the Fick’s law and it is approximately given by the exponential relation:

(8) CP(r)=CP(0)⋅exp(−rl)

where “*r*” is the distance from the plasma source (Figure 14a) and “*l*” is the charge diffusion length.

**Figure 15:**
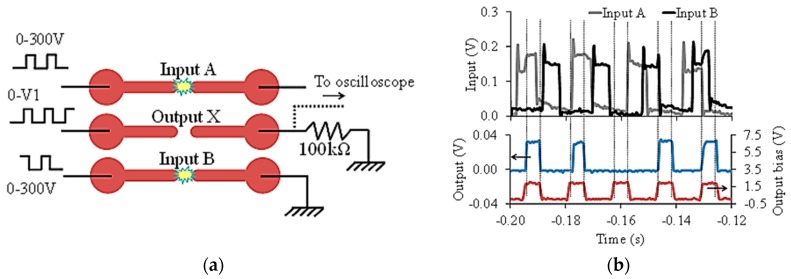
The electrode configuration used to implement OR logic is shown in (**a**) and is similar to the experiment shown in Figure 14c. The response of the OR gate is shown in (**b**). The output is valid only when output bias is high. The amplitude of output bias pulse is 195 V in this case. Both the figures are reproduced from [35].

We have reported many logical gate operations using plasma interconnects in the past [25,33,34,35]. Here we discuss the OR logic operation that was implemented using the arrangement shown in Figure 15a. To realize the OR operation, we biased the central device (X) at 195 V just below its breakdown voltage. When either of its adjacent devices (A and B) are turned ON they produce enough excess electrons for the central device X to turn on (Figure 15b). After X is turned ON, it does not turn OFF if the inputs A and B are turned OFF because the plasma at X remains self-sustained. X can be turned OFF by turning down its bias voltage significantly or turning it OFF.

**Figure 16:**
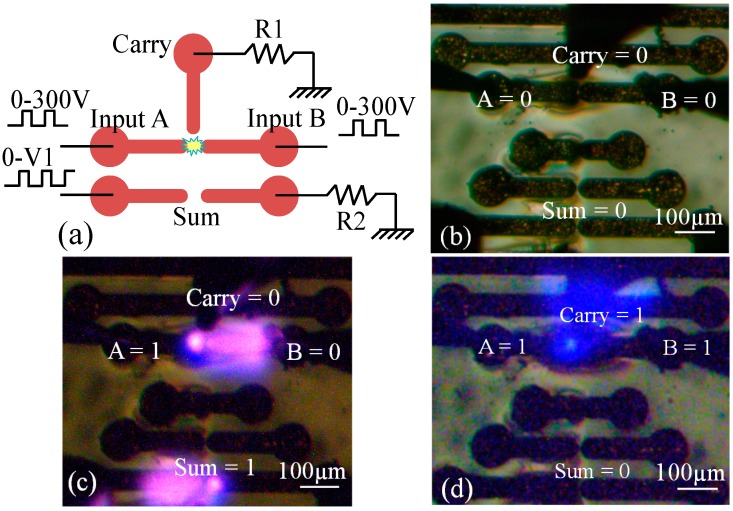
(**a**) Schematic representation of the electrode setup for 1-bit half-adder. State of the Sum and Carry bits are represented by plasma on their corresponding electrodes for the input states (A, B = 0), (A = 1, B = 0) and (A, B = 1) in (**b**–**d**), respectively. All figures are reproduced from [35].

A 1-bit half-adder circuit was also realized as shown in Figure 16a. The operation of the device for the “Sum” bit is the same as that of XOR.

An advantage of implementing logic gates using plasma interconnect is in the reduction of number of active switching elements. For example, AND and OR gates implemented using complementary metal oxide semiconductor (CMOS) require four transistors each while XOR requires eight transistors. Implementing these gates with plasma-connected circuits requires only 2–3 μPDs. μPD devices reported here were to show the feasibility of realizing functional plasma gates and sub-circuit.

## 6. Distributed Micro Plasma Devices

The above examples show the possibility of constructing microplasma devices with gate electric field or gate current control of the plasma current and voltage. In the above devices the spatially confined plasma interacted with the gate field over a small and “point like” region comparable to the electron mean-free path (0.5 μm). It is also possible to devise distributed plasma devices where the interaction between the plasma and fields occur over many 100 s of micrometers; large compared to the electron mean free path.

**Figure 17:**
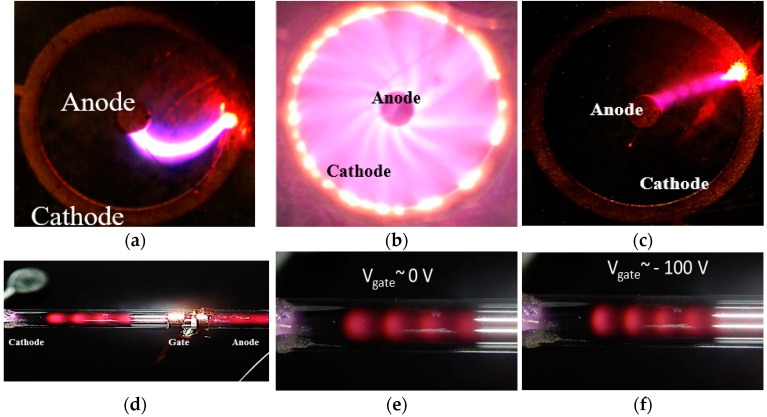
(**a**) A coaxial pin-ring structure with a plasma arc that is bent by applying a perpendicular magnetic field; (**b**) the same device with the plasma arc un-pinned and rotating around the ring in the presence of the external magnetic field [29,39]; (**c**) striations are observed in the ring devices; and (**d**–**f**) a linear extended plasma device with striations controlled using a gate electrode. The rings are around 1 cm in diameter. The linear device is 4 cm long.

Figure 17a shows a circular plasma device where a plasma arc generated between an inner pin and an outer annular ring responds to an applied magnetic field through the Lorentz force. If the plasma arc is “pinned” because of imperfections in the ring or the central pin, it deflects and becomes curved in the presence of a perpendicular magnetic field (Figure 17a). In this device, helium was used in 1 Atmosphere and the magnetic fields as low as 1 μT could deflect the plasma arc. If the plasma arc is free to rotate and it is not “pinned”, it does so as shown in Figure 17b. This ring was used as a magnetometer and can be used as a rotary switch and as an amplifier. Figure 17c shows striation in helium plasma at 100 Torr that are spatially modulated using a voltage applied to the gate electrode. Striations are charged regions and their spatial modulation gives rise to modulation of the local fields in the plasma [36,37].

**Figure 18:**
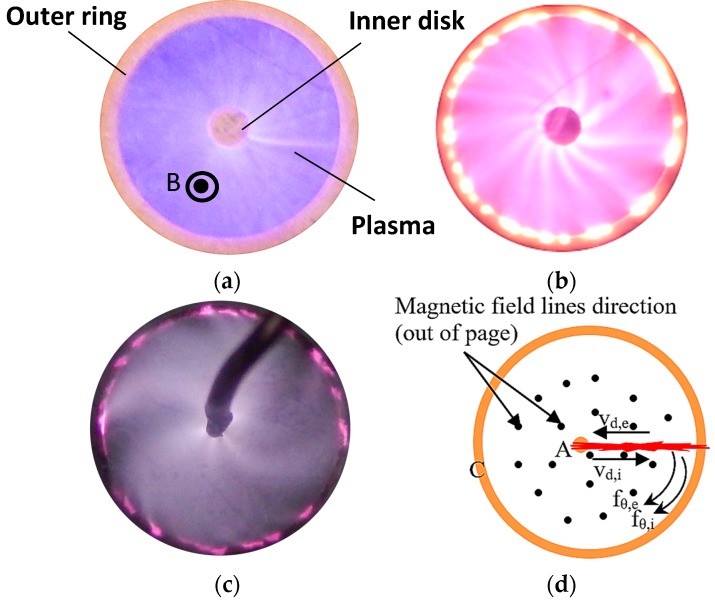
Rotating glow discharges at atmospheric pressure for: (**a**) Helium; (**b**) Neon; and (**c**) Argon; (**d**) Schematic of different components of Lorentz forces exerted on the glow discharge. The rotations of Neon and Argon glow have some interesting spatial feature as seen from the “spokes” in their rotations [29,39]. B in (a) indicates the magnetic field, A in (d) indicates anode, and C in (d) indicates cathode.

Striations, believed to be ionization waves [36,37], were also observed and controlled inside the MPDs as can be seen in Figure 18. Different gases show different ionization patterns inside the co-axial pin-ring device, as shown in Figure 18a–c [29,39]. In all cases, both the ionic and electronic currents are deflected in the same direction as schematically, as shown by *f_θ_*_,*I*_ and *f_θ_*_,*e*_ in Figure 18d.

Similar to electron beam traveling tube amplifiers, it is also possible to realize plasma traveling tube amplifiers [29,40]. The main motivations for using plasmas instead of electron beams are: (a) thermionic emission required in e-beam generation can be replaced with gas ionization; (b) electrostatic lenses and magnetic focusing structures can be eliminated or reduced in complexity since plasma is self-focusing; and (c) larger acceleration fields can be used since plasma is quasi-neutral. It is interesting to note that the plasma pressure can be varied to yield an electron beam in the limit of device critical dimension becoming comparable or smaller than the electron mean-free path. It is also possible to increase interactions between a laser and the plasma using its space charge fields at its boundaries. This “wake-field” interactions are used in 100 GeV chip-scale electron accelerators.

**Figure 19:**
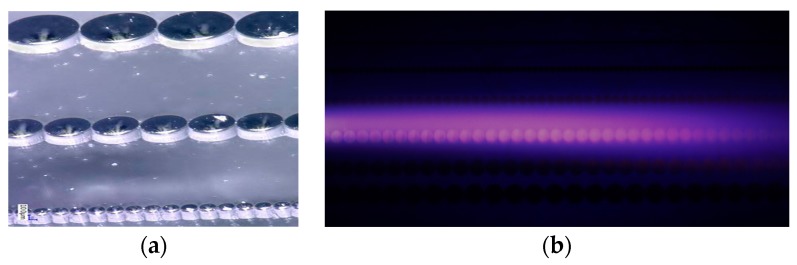
(**a**) SEM of quartz dielectric coupled resonators where the terahertz wave is guided; and (**b**) optical image of the quartz resonators under a helium plasma [40]. The scale bar is 100 μm.

Figure 19a shows SEM of a periodic structure that was etched using deep reactive ions in quartz. Figure 19b shows atmospheric helium plasma superimposed on the quartz array to interact with the terahertz signal traveling in the quartz periodic structure. The periodic quartz array constitutes a “slow” wave structure enabling efficient interaction between the plasma and the terahertz signal. The interaction can be used to amplify the terahertz wave or to accelerate electrons in the plasma using the terahertz signal. We have designed, fabricated and tested many terahertz microplasma traveling wave amplifiers reported elsewhere [29,39,40].

## 7. Material Requirements

Materials used in MPDs determine the reproducibility of their characteristics and ultimately their useful lifetime [28]. MPDs that operate with dc voltages are in particular prone to electrode-related failures due to sputtering of their electrode materials. Thus, it is important to use metals that have very low sputtering yields and very high thermal conductivity.

The dielectric regions in MPDs should also be able to withstand large thermal gradients and large electric fields. High thermal conductivity materials such as quartz last longer than glass but it is much more difficult to etch.

The insensitivity of plasma to high temperatures and ionizing radiation are advantageous to the operation of the MPDs in these harsh environments. The partially ionized plasma in the microplasma transistor typically has temperatures in the range of several hundred degrees Celsius depending on the gas pressure, plasma density and the current density through the plasma. The gaseous state of the “active” region in the transistor is unaffected by the elevated temperatures in the plasma. However, the electrodes that generate and sustain the plasma are affected by elevated temperatures in different ways. The most obvious effects are softening, thermal expansion and development of residual stress along the cross section. Apart from the high temperature, plasma is also equipped with energetic ions that can sputter the electrode material that becomes more severe at elevated temperatures. Considering these factors, one can list several desirable properties that should be expected in a material qualifying for the electrodes. Following are some important properties of a potential electrode material that will ensure its longevity:

- High melting temperature
- High density to minimize the sputtering damage
- Good thermal conductivity to carry the heat away from the electrodes
- Low thermal expansion coefficient to prevent expansion of the electrode layer

High thermal conductivity and low thermal expansion coefficient also prevent buildup of residual stress along the cross section by avoiding temperature gradients along the cross section. Besides these, there is an additional requirement that can be understood by considering the structure of the microplasma transistor. For the current discussion it is sufficient to mention that the electrode(s) are in the form of a beam supported by an anchor. For reproducible switching, the electrodes have to remain fixed in their position as the switching (breakdown) voltage is sensitive to the mutual positioning of the electrodes. Thus, it is desirable to use a material with a high elasticity that does not comply easily, which is reflected by its Young’s modulus. Based on all these requirements, a figure of merit (FOM) was defined as [28]:

(9) $$\mathrm{FOM}=\frac{\gamma \times 10^{9}}{E\times \rho \times M\times \alpha }$$

where γ is the thermal expansion coefficient, E is the Young’s modulus of elasticity, ρ is the material density, M is the melting point in °C and α is the thermal conductivity. A good electrode is expected to have a low FOM. Table 1 lists the properties of some common materials used in microfabrication along with their calculated FOM. Based on these FOMs, tungsten seems to be the best choice. Tungsten is a good refractory material and is well known for its physical hardness. In our devices, copper electrodes outperformed tungsten probably because of their higher thermal conductivity [24,33,34,35].

In summary, we discussed three terminal microplasma devices with MOSFET-like gate structures and devices with co-planar control electrodes. We showed that the gate field effect can be used to affect the plasma parameters and control the current in theses MPDs. Both analog and digital operation of these MPDs were demonstrated and discussed. Finally, we briefly discussed distributed MPDs for sensing and interactions with terahertz signals. Lower voltages in our microplasma devices were achieved by taking advantage of the ion-enhanced field emission that through Fowler–Nordheim electron tunneling reduces the breakdown voltage in small gap devices. In our devices, the gate (control) electrode was used to change the concentration of the secondary electrons that in turn changed the breakdown voltage and hence the plasma current when the drain-source voltage was fixed. We also discovered that boundaries are quite important in miniature plasma devices and they soak up electrons even when they are covered with dielectrics. RF plasma devices lasted longer than DC devices due to reduced electrode material sputtering. RF plasma devices operating at 10 GHz were demonstrated.

## 8. Conclusions

We discussed a new class of micro-fabricated plasma devices operating near atmospheric pressure and demonstrated metal-oxide plasma field effect transistors and a range of related devices used to characterize micro-plasmas.

## Figures and Tables

**Table 1:** Properties of some common materials used in microfabrication considered as potential candidates for the MOPFET electrodes. Tungsten is the material of choice due to its low FOM. Experimentally, copper also worked well because of its high thermal conductivity [25].

Material	*E* [GPa]	*ρ* [g/cm^3^]	*M* [°C]	*α* [W/mK]	*γ* [10^−6^/K]	FOM
Aluminum	70	2.7	660	237	24	812
Chromium	279	7.19	1907	93.9	4.9	14
Copper	128	8.96	1084	401	16.5	33
Platinum	168	21.45	1768	71.6	8.8	19
Poly-Si	188	2.3	1414	149	2.6	29
Tantalum	186	16.6	2996	57.5	6.5	12
Titanium	166	4.5	1668	21.9	8.6	315
Tungsten	411	19.25	3422	175	4.5	1
